# Statins’ Regulation of the Virulence Factors of *Helicobacter pylori* and the Production of ROS May Inhibit the Development of Gastric Cancer

**DOI:** 10.3390/antiox10081293

**Published:** 2021-08-16

**Authors:** Ting-Yu Lin, Wen-Hsi Lan, Ya-Fang Chiu, Chun-Lung Feng, Cheng-Hsun Chiu, Chia-Jung Kuo, Chih-Ho Lai

**Affiliations:** 1 School of Medicine, College of Medicine, Chang Gung University, Taoyuan 33302, Taiwan; b0902110@cgu.edu.tw (T.-Y.L.); b0902063@cgu.edu.tw (W.-H.L.); yfchiu@mail.cgu.edu.tw (Y.-F.C.); chchiu@cgmh.org.tw (C.-H.C.); 2 Research Center for Emerging Viral, Infections Department of Microbiology and Immunology, Graduate Institute of Biomedical Sciences, Chang Gung University, Taoyuan 33302, Taiwan; 3 Department of Medical Laboratory, Chang Gung Memorial Hospital at Linkou, Taoyuan 33305, Taiwan; 4 Division of Gastroenterology and Hepatology, Department of Internal Medicine, China Medical University Hsinchu Hospital, Hsinchu 30272, Taiwan; d6604@mail.cmuhch.org.tw; 5 Department of Internal Medicine, Department of Medical Research, School of Medicine, China Medical University and Hospital, Taichung 40447, Taiwan; 6 Molecular Infectious Disease Research Center, Department of Pediatrics, Chang Gung Memorial Hospital at Linkou, Taoyuan 33305, Taiwan; 7 Chang Gung Microbiota Therapy Center, Chang Gung Memorial Hospital at Linkou, Taoyuan 33305, Taiwan; 8 Department of Gastroenterology and Hepatology, Chang Gung Memorial Hospital at Linkou, Taoyuan 33305, Taiwan; 9 Department of Nursing, Asia University, Taichung 41354, Taiwan

**Keywords:** statin, gastric cancer, cholesterol, reactive oxygen species, *Helicobacter pylori*

## Abstract

Conventionally, statins are used to treat high cholesterol levels. They exhibit pleiotropic effects, such as the prevention of cardiovascular disease and decreased cancer mortality. Gastric cancer (GC) is one of the most common cancers, ranking as the third leading global cause of cancer-related deaths, and is mainly attributed to chronic *Helicobacter pylori* infection. During their co-evolution with hosts, *H. pylori* has developed the ability to use the cellular components of the host to evade the immune system and multiply in intracellular niches. Certain *H. pylori* virulence factors, including cytotoxin-associated gene A (CagA), vacuolating cytotoxin A (VacA), and cholesterol-α-glucosyltransferase (CGT), have been shown to exploit host cholesterol during pathogenesis. Therefore, using statins to antagonize cholesterol synthesis might prove to be an ideal strategy for reducing the occurrence of *H. pylori*-related GC. This review discusses the current understanding of the interplay of *H. pylori* virulence factors with cholesterol and reactive oxygen species (ROS) production, which may prove to be novel therapeutic targets for the development of effective treatment strategies against *H. pylori*-associated GC. We also summarize the findings of several clinical studies on the association between statin therapy and the development of GC, especially in terms of cancer risk and mortality.

## 1. Introduction

Gastric cancer (GC) is a global health burden with more than a million new cases diagnosed in 2020, and the mortality rate is only surpassed by that of lung and liver cancers [1]. GC progresses in multiple stages including superficial gastritis, atrophic gastritis, intestinal metaplasia, dysplasia, and finally GC [2]. Lifestyle and behavior are exogenous risk factors closely associated with the development of GC [3]. *Helicobacter pylori* infection is one of the most important causative agents of GC and is associated with higher risk in at least 80% of cases [4]. *H. pylori* is a gram-negative, microaerophilic, spiral bacterium that generally colonizes the stomach. Usually acquired at a young age and without therapeutic means of eradication, *H. pylori* can colonize the host’s stomach for their entire lifetime [5]. Persistent *H. pylori* infection accumulates oxidative stress and prolongs the inflammation in gastric mucosa, resulting in the development of GC [6].

Surgical resection is a common curative treatment for GC, whereas endoscopic resection is an option for patients with small, well-differentiated early-stage tumors [7]. Adjuvant and neoadjuvant therapies, such as chemotherapy and radiotherapy are recommended in combination with surgery [8]. However, surgical treatments are invasive, and patients often suffer from surgical trauma, immunosuppression, postoperative morbidity, and mortality [9]. The management of metastatic GC includes cytotoxic therapy and chemotherapy. Although the former is associated with a high frequency of toxic effects, the latter appears to have a limited effect on overall mortality. As many patients with early-stage GC are asymptomatic, diagnosis is usually made when the tumor has metastasized and surgical resection no longer remains a curative treatment option. Therefore, preventive measures for GC in high-risk individuals are worth investigating.

Statins are inhibitors of 3-hydroxy-3-methylglutaryl-coenzyme A (HMG-CoA) reductase, which is commonly prescribed for primary and secondary cardiovascular disease prevention, and they have attracted attention as a popular candidate for chemoprevention [10]. By reducing low-density lipoprotein cholesterol (LDLC) levels in blood circulation, statins effectively lower the risk of atherosclerotic cardiovascular diseases [11,12]. Mounting evidence has shown reduced cancer incidence and mortality in patients who were prescribed statins [13,14,15,16,17,18,19]. In terms of adverse effects, it has been suggested that statins are potentially diabetogenic (causing diabetes mellitus) and increase the incidence of hemorrhagic strokes [20,21]. Myopathy, a condition defined as muscle pain and tenderness, has also been reported as an adverse effect [20]. However, a study found that statin use was not correlated with myopathy [22], and did not increase the risk of hemorrhagic stroke in patients without prior incidence of stroke [23].

Although eradication of *H. pylori* using antibiotics is a viable method to reduce the risk of GC, it may have consequences such as an increase in antibiotic resistance rates, short-term alterations in gut microbiota, and increased risks of asthma and allergies [24,25]. Because statins primarily target virulence factors, they do not affect the extracellular survival of *H. pylori*, they only reduce the intracellular burden of infected cells [26]. In addition, statins have been reported to decrease GC risk independent of *H. pylori* status [27,28]. As such, statins cause little changes to the gut microbiota, making it a more ideal candidate for chemoprevention than eradication therapy of *H. pylori*. Overall, statins are not associated with clinically significant side effects, underscoring their potential as risk-reducing agents for GC.

By drawing links between *H. pylori* virulence factors and statin actions, we unravel the mechanisms by which statins may be employed to reduce GC incidence in patients with *H. pylori* infection. We also discuss previous database analyses to summarize the effects of statins on GC prevention in clinical settings, and review the prospects for statins as potential chemopreventive medications.

## 2. *H. pylori* Virulence Factors Usurp Cholesterol and Lead to GC Development

*H. pylori* infection induces sustained inflammation, an event that has long been linked to cancer development [29,30]. In addition to the host immune response, *H. pylori* virulence factors promote sustained inflammation, thus maintaining a microenvironment rich in cytokines/chemokines, reactive nitrogen species (RNS), and reactive oxygen species (ROS) that can destabilize normal cellular homeostasis [29]. Chronic inflammation exhausts resident gastric stem cells [31] and leads to the recruitment of bone marrow-derived cells that are predisposed to improper differentiation, resulting in metaplasia and dysplasia [30]. *H. pylori* virulence factors play an important role in inducing inflammatory responses and promoting pro-tumorigenic activities.

Vacuolating cytotoxin A (VacA) is a virulence factor well known for its ability to cause vacuoles that possess the hallmarks of late endosomes and early lysosomes in host cells [32]. Although the role of these vacuoles in GC remains unclear, it is posited that VacA-induced vacuoles disrupt normal membrane trafficking at or near late endosomes [33]. Subsequently, VacA induces autophagy and impairs transient receptor potential membrane channel mucolipin 1 (TRPML1), a key regulator of the endolysosomal pathway [34]. Consequently, the inhibition of lysosomal function or preventing the fusion of autophagosomes with lysosomes promotes bacterial resistance and multiplication of autophagosomes, which is crucial for persistent bacterial infection and induction of gastric carcinogenesis [35,36,37]. VacA triggers the production of proinflammatory cytokines, such as tumor necrosis factor (TNF) and interleukin (IL)-6, which promote the expression of cyclooxygenase*-*2 (COX-2) in T cells, macrophages, and neutrophils [38]. In addition to participating in proinflammatory events, COX-2 catalyzes a key step in the production of prostaglandin, a group of lipids known to play a role in tumorigenesis [29].

Another virulence factor is cytotoxin-associated gene A (CagA), which is encoded by the *cag-*pathogenicity island (*cag*-PAI) and is the most extensively studied virulence factor of *H. pylori* for its cancer-causing actions. *H. pylori* strains carrying *cagA* (referred to as *cagA*-positive strains) were significantly more correlated with the exacerbation of gastric conditions and gastric adenocarcinoma than *cagA*-negative strains [39]. The *cag*-PAI gene encodes a type IV secretion system (T4SS), a protein complex that is critical for the translocation of CagA across the membrane. Following its translocation into gastric epithelial cells, CagA is localized to the inner membrane and immediately phosphorylated by members of the Src family kinase [40]. Subsequently, CagA binds to the SH2 domain-containing tyrosine phosphatase SHP2, which potentiates a downstream cascade of Erk/MAPK activity and affects the regulation of cellular proliferation, growth, and morphology, leading to deregulation of phase progression from G1 to S [41,42] and to an increase in the expression of proto-oncogenes *c-fos* and *c-jun* [43]. In addition, CagA phosphorylation induces activation of the transcriptional factor nuclear factor-kappa B (NF-κB) and production of the cytokine IL-8 in gastric epithelial cells [44,45]. NF-κB regulates many genes whose products are involved in angiogenesis, anti-apoptotic pathways, metastasis, enhanced cell cycle progression, and cytokine production. IL-8 has been reported to have an angiogenic role in several types of cancer [29].

Cholesterol-α-glucosyltransferase (CGT), encoded by the type I capsular polysaccharide biosynthesis protein J gene (*capJ*) catalyzes the glucosylation of cellular cholesterol into cholesteryl glucosides [46]. Cholesterol glucosylation dampens *H. pylori* phagocytosis and T-cell activation, leading to bacterial immune evasion [47]. Furthermore, CGT increases autophagosome formation, which enhances *H. pylori* survival in host macrophages by providing an intracellular niche [48,49]. This process also reduces autophagosome-lysosome fusion, which is a key step in eliminating intracellular pathogens. Furthermore, cholesterol glucosylation improves *H. pylori*-host cell binding and reorganizes lipid raft membranes, thereby promoting T4SS functions such as CagA translocation/phosphorylation. This activates NF-κB to promote IL-8 production, thereby aggravating inflammation [50].

## 3. Interplay between *H. pylori* and ROS Production to Induce Gastric Carcinogenesis

*H. pylori* CagA translocated in the cells is crucial for inducing the production of a significant amount of ROS, which is involved in the enforcement of cell cycle progression and acceleration of cell proliferation [51]. In addition, the accumulation of ROS increases oxidative stress, which damages mitochondrial DNA (mtDNA) and nuclear DNA, leading to gastric carcinogenesis [52]. Apart from ROS, nitrosative stress is another key mediator of *H. pylori* infection. Nitric oxide (NO) derived from inducible nitric oxide synthase (iNOS) is responsible for bacteria-induced inflammatory responses [53]. *H. pylori* elicits NO production in macrophages and gastric epithelial cells, which convert l-arginine to l-citrulline using iNOS [54,55]. In GC patients with *H. pylori* infection, iNOS expression is higher than that in *H. pylori*-negative individuals [56]. iNOS deficiency lowered NO production by iNOS and markedly reduced *H. pylori*-associated GC in mice [57], indicating that overexpression of iNOS and sustainable NO levels contribute to *H. pylori*-induced stomach carcinogenesis.

VacA also plays a role in ROS generation. In a gastric epithelial model, *H. pylori* with CagA^+^/VacA^+^-induced ROS production and mtDNA mutations were significantly higher than those in a VacA-mutant strain [58]. Furthermore, infection with the VacA^+^ strain elevates SQSTM1/p62 aggregation and disrupts autophagy to increase ROS expression in gastric epithelial cells, which may accelerate carcinogenesis [35]. Biopsies from patients with Crohn’s disease showed that *ATG16L1* with the T300A mutation increases susceptibility to *H. pylori* infection, indicating that the *ATG16L1* genotype modulates autophagy responses to VacA [59]. Notably, autophagy and CagA can be degraded by VacA by reducing intracellular glutathione levels, leading to enhanced ROS accumulation and Akt phosphorylation, resulting in GC development [60]. The mechanism by which VacA and CagA decrease autophagy may provide a unique strategy for persistent *H. pylori* colonization in the stomach. These findings support the view that elevated ROS production due to *H. pylori* infection enhances DNA damage and prevents DNA repair mechanisms from functioning properly, thereby contributing to gastric carcinogenesis [6].

*H. pylori* has evolved to elicit detrimental effects in cells while dampening the host’s defenses using strategic mechanisms [61]. *H. pylori* arginase competes with cellular iNOS for the substrate l-arginine, which reduces NO production [62]. In addition, arginase II produced by macrophages suppresses *H. pylori*-induced NO production by inhibiting iNOS expression [63]. Reducing l-arginine availability decreases *H. pylori*-stimulated iNOS expression and NO levels [64]. In parallel, VacA inhibits the expression of integrin-linked kinase and endothelial nitric oxide synthase, thereby decreasing ROS production in macrophage/monocyte-lineages [65]. These findings indicate that *H. pylori* exploits host factors to orchestrate ROS generation, resulting in simultaneous damage to cells and immune evasion.

## 4. Statins Lower GC Risk by Reducing *H. pylori* Survival and Inhibition of Virulence Factor Actions

Statins are competitive inhibitors that block the conversion site of HMG-CoA reductase to prevent substrate access and effectively inhibit the conversion of HMG-CoA into mevalonic acid [66]. With a reduced level of mevalonic acid, the cholesterol synthesis pathway is interrupted in the liver [67]. This triggers the production of microsomal 3-hydroxy-3-methylglutaryl-CoA reductase and cell-surface low-density lipoprotein (LDL) receptors, which assist in lowering the level of circulating LDL in the bloodstream to 20–55% [68]. By inhibiting mevalonate synthesis, statins inhibit the production of mevalonate-derived intermediates, which are involved in the post-translational modification of proteins crucial for intracellular signaling, cell growth, and cellular differentiation [69,70].

**Figure 1:**
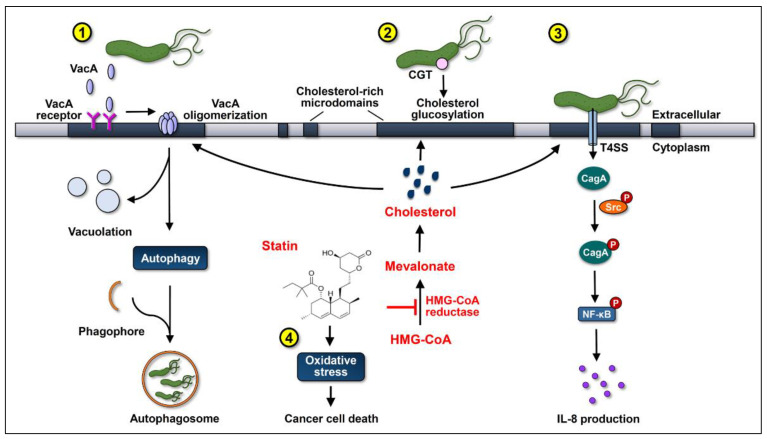
Statins inhibit cholesterol synthesis by interrupting the conversion of 3-hydroxy-3-methylglutaryl-coenzyme A (HMG-CoA) to mevalonate, consequently suppressing the actions of the virulence factors of *Helicobacter pylori* and increasing oxidative stress to promote gastric cancer cell death. (1) After cholesterol-dependent internalization, vacuolating cytotoxin A (VacA) induces vacuolation in the late endosomal compartments in host cells. Subsequently, VacA promotes autophagy and interferes with lysosomal functions, which sequesters *H. pylori* in intracellular niches to multiply. (2) Cholesterol-α-glucosyltransferase (CGT) catalyzes the glucosylation of cholesterol, contributing to immune evasion and intracellular survival of *H. pylori*. (3) Cytotoxin-associated gene A (CagA) translocation/phosphorylation depends on the coalescence of cholesterol-rich microdomains and glucosylated cholesterol, which in turn activates the nuclear factor-kappa B (NF-κB) and enhances the production of interleukin (IL)-8. The use of statins reduces the cellular cholesterol levels, inhibits the internalization of VacA, decreases the glucosylation of cholesterol, and attenuates the actions of CagA, thereby alleviating the *H. pylori*-induced pathogenesis. (4) Statins increase the production of reactive oxygen species (ROS) to enhance oxidative stress, thereby synergistically promoting their anti-cancer properties.

Simvastatin is a class of statins that has been reported to reduce the level of cellular cholesterol in macrophages and gastric epithelial cells [13,26]. As both CagA translocation and phosphorylation depend on adequate cholesterol levels, treatment with simvastatin significantly reduces CagA translocation into gastric epithelial cells [13], which may in turn attenuate CagA-induced oncogenesis (Figure 1). In addition, treatment of cells with cholesterol-depleting agents reduces VacA internalization and cholesterol likely plays an important role in VacA entry into cells [71]. It is possible that statins may inhibit VacA internalization by gastric epithelial cells, subsequently inhibiting its pro-tumorigenic effects. Our recent study further demonstrated that the cholesterol-reducing effect of statins interrupts the cholesterol-dependent cellular evasion strategies of *H. pylori* mediated by CGT and reduces the bacterial load in macrophages [26]. Most importantly, several studies have reported a wide range of actions performed by statins that could reduce cancer incidence besides the inhibition of the internalization of *H. pylori* virulence factors. These include reduction in the plasma concentration of inflammatory cytokines, attenuation of proliferative response, and modulation of immune responses [69].

## 5. Statins Modulate MicroRNAs and Exosome Levels

MicroRNAs (miRNAs) are short, noncoding segments of RNA involved in the regulation of gene expression at the post-translational level [72]. Zambrano et al. investigated the effects of low dose short-term statin treatment and found that statin affects the expression of certain miRNAs [73]. Although simvastatin did not significantly impact the expression of the 86 miRNAs studied, it appeared to up-regulate several miRNAs that are involved in tumor progression in subjects who exhibited a high reduction in the level of LDLC. In addition, atorvastatin was associated with poor expression of some miRNAs studied, and it possibly downregulated the level of miRNA-33, a miRNA that reduces fatty acid metabolism and cholesterol transport [73,74].

Other than miRNAs, exosomes have been reported to lower cholesterol levels through the use of statins. Exosomes are extracellular vesicles that function in cell-to-cell signaling, and simvastatin was found to repress exosomal formation and secretion [75]. As cholesterol is an integral component of exosomal membrane, it is natural to attribute this outcome to its cholesterol-lowering effect; however, the results of the study revealed that alternative pathways may be at play instead. Because exosomes are thought to have proinflammatory activities, inhibition of exosome biogenesis and secretion may reduce inflammation, which suggests a possible chemopreventive mechanism of simvastatin.

In addition to regulating cholesterol synthesis, the virulence factors of *H. pylori*, and ROS responses, it is possible that statins reduce the risk of *H. pylori*-associated GC via the regulation of miRNAs and exosomes. For example, miRNA-146 and miRNA-155 are induced after *H. pylori* infection to regulate inflammation [76,77]. Another miRNA, let-7b is reduced in GC cells in a CagA-dependent manner, which may lead to the downregulation of TLR4 [78]. Additionally, miRNA-451, which inhibits the macrophage migration inhibitory factor (MIF) and functions as a tumor suppressor, is also downregulated after *H. pylori* infection in GC cells [79]. miRNA-29a is downregulated in GC cells to promote cell cycle progression and proliferation [80]. However, statins are known to reverse the functions of these miRNAs, suggesting that these drugs may be useful for treating GC caused by *H. pylori*. Meanwhile, simvastatin is known to suppress exosome formation [75], which allows cells to deliver various miRNAs and CagA for the pathogenesis of *H. pylori*.

## 6. Cholesterol-Independent Beneficial Effects of Statins in Cancer Therapy

In addition to their cholesterol-lowering function, statins exert pleiotropic therapeutic effects, which have been demonstrated to reduce the risk of several types of cancer. Statins possess anti-cancer properties, mainly by virtue of their high NO production, which is essential for tumor cytotoxicity [81]. For example, fluvastatin and simvastatin have been shown to be cytotoxic to human breast cancer cells by elevating iNOS activity and inhibiting geranylgeranylation [82]. Activation of iNOS increases NO levels, which arrests the cell cycle in the G1 phase and downregulates cyclin D1, leading to synergistically enhanced statin-induced cancer cell death [83]. In addition, lovastatin and simvastatin increase mitochondrial membrane potential (ΔΨ) and modulate mitochondrial metabolism in several cancer cells independent of cholesterol content [84]. Nonsteroidal anti-inflammatory drugs (NSAID) have been found to cause gastropathy, which was attributed to redox imbalance [85]. Notably, statins exert a protective effect against NSAID-induced lesions by increasing NO production and prostaglandin expression [86]. Overall, the NO-mediated proapoptotic, tumoricidal, and antiproliferative effects of statins confer anti-cancer properties.

Although ROS is activated as the cellular defense mechanism against bacterial invasion, *H. pylori* has evolved a variety of strategies, including antioxidant and DNA repair enzymes for facilitating its long-term survival within host cells [63,87]. However, persistent *H. pylori* infection enhances genetic instability and high mutations are generated during the repair processes, leading to gastric carcinogenesis [88]. In contrast, the excessive ROS induced by statins is irreversible, which leads to the accumulation of these ROS/RNS at toxic levels and results in profound cell death for therapeutic benefits [89,90].

## 7. Statin Use Reduces GC Risk

Statins were originally used to lower cholesterol levels to prevent cardiovascular disease. In addition to cholesterol restriction, statins are potential drugs for cancer therapy. To investigate the direct relationship between statins and anti-cancer activity, its effect on several types of cancers has been analyzed [91]. The results showed that statins induce cancer cell death by triggering the apoptotic pathway, not only in cell models but also in murine GC xenografts [92,93]. These findings suggest that statins may possess anti-cancer activity; however, very few clinical studies have reported that statins are anti-cancer drugs. This review further discussed the role of statin use in GC prevention and treatment (Table 1 and Table 2, respectively). Chiu et al. conducted a population-based case-control study in Taiwan, which showed a lower risk of GC in statin users than in non-users (OR = 0.68, 95% CI = 0.49–0.95) [18]. Notably, a dose-dependent effect was observed between statin use and GC risk. Lee et al. conducted a clinical study to examine the association between statin use and GC by analyzing patients with diabetes [27]. Their results showed that prescription for any statins exhibited a significant inverse association with GC. Additionally, the duration of statin use was positively correlated with a reduction in the risk of GC in patients with diabetes. These anti-GC effects warrant further research on the potential clinical use of statins.

*H. pylori* infection is closely associated with GC incidence [4,99,100]. Membrane cholesterol-rich microdomains provide specific regions for *H. pylori* virulence factor-induced pathogenesis and GC development [47,101,102]. Statins may reduce cholesterol levels and attenuate bacterial virulence factor actions, which may alleviate *H. pylori*-associated diseases, making them a plausible therapeutic option to treat *H. pylori*-induced GC. Our recent study reported that statin treatment reduced CagA translocation/phosphorylation levels and mitigated *H. pylori*-induced pathogenesis [13]. These results suggest that statins can decrease several pathogenic effects caused by *H. pylori* virulence factors. We then conducted population-based case-control studies by analyzing the Taiwan National Health Insurance Research Database and demonstrated that patients who received simvastatin exhibited a remarkably low incidence of GC [13]. GC risk reduction is especially significant in patients with *H. pylori* infection compared to that in statin non-users (adjusted OR = 0.25, 95% CI = 0.12–0.50). A similar trend was observed with the use of other types of statins. In addition to the type of statin prescribed, the defined daily dose is a factor related to the efficacy of GC risk reduction. These results demonstrate that statin use significantly reduces the incidence of GC, particularly in patients with *H. pylori* infection. However, the mechanism by which statins lower the risk of *H. pylori*-related GC requires further investigation.

Conversely, research by Toyoda’s group indicated that pitavastatin failed to suppress GC in murine models [94]. This study examined the relationship between pitavastatin and *H. pylori*-associated gastric carcinogenesis by adding pitavastatin to the diet of *H. pylori*-infected Mongolian gerbils. Compared to the control group, the incidence of *H. pylori*-associated gastric adenocarcinomas was not reduced in pitavastatin-treated mice. Serum total cholesterol also increased in the experimental groups treated with pitavastatin compared to that in the untreated controls. These results indicate that pitavastatin is ineffective in suppressing *H. pylori*-induced GC in murine models.

Although *H. pylori* infection is an important causative agent of GC, the possibility of developing GC still exists [103]. Statins interfere with *H. pylori* infection and suppress the delivery of virulence factors to the cells [13,26]. *H. pylori* infection could be a confounding factor that may cause bias in clinical studies. To eliminate the confounding effect of *H. pylori* status, Cheung et al. investigated the effect of statins in *H. pylori*-eradicated GC by analyzing the clinical data and reporting system in Hong Kong [96]. Competing risk regression with propensity score matching revealed that statin prescription was related to reducing the risk of GC in patients who had received *H. pylori* eradication therapy. The risk difference was 2.6 times lower in GC cases (95% CI = 1.56–3.12) per 10,000 person-years in statin users than in statin non-users. These results support the use of statins as chemopreventive agents for the treatment of GC in *H. pylori*-eradicated patients.

Spence et al. analyzed the relationship between statin use and GC mortality in England [14]. Two independent databases, the UK Clinical Practice Research Datalink in England and the Prescribing Information System in Scotland were investigated. These two databases recorded statin prescription and death information identified from the national mortality records. Combined cohorts and hazard ratio (HR) analysis showed that patients with GC who received statins exhibited a reduction in mortality (adjusted HR = 0.83, 95% CI = 0.74–0.92). Cancer-specific mortality was also reduced for patients who were prescribed statins before diagnosis with GC (adjusted HR = 0.91, 95% CI = 0.84–0.98). However, the doses of statins used in the treatment of patients with GC were not analyzed.

The association between statin use and lower GC risk remains elusive, and some discrepancies have emerged. A study by Cho et al. showed that statins were able to reduce mortality in GC but failed to decrease its incidence [98]. These findings are inconsistent with results from previous systematic reviews and meta-analyses, which may be because different study populations and settings were analyzed [17]. Although recent research has focused on using statins as an agent in GC treatment, whether statins can be used in the prevention of GC requires further supporting evidence. Further research that includes a large cohort with different populations should be conducted to clarify whether the clinical use of statins in the treatment of GC is feasible.

## 8. Conclusions and Perspectives

Most studies conducted in Western and Eastern countries to investigate the relationship between statin use and the development of GC have reported similar results, which suggest that the use of statins can reduce the risk of GC. However, most of these studies were still in the preclinical stage or were only conducted in the form of database analysis. The mechanisms by which statins inhibit *H. pylori* infection, especially the link between statin treatment and the manipulation of autophagy to eliminate *H. pylori*, remain unclear and need to be investigated further [26]. Moreover, the mechanisms by which statins increase ROS levels and regulate oxidative stress to promote GC cell death need to be elucidated. The current information from in vivo studies is insufficient, and further investigations should be conducted to determine whether statins can potentially be used for the treatment of GC.

Some studies have reported that the use of statins is associated with reducing the risk of other types of cancer. Data mining conducted using databases from the Food and Drug Administration (FDA) Adverse Event Reporting System and the Japan Medical Data Center, examined the association between the use of statins and different types of cancer, including colorectal, lung, pancreatic, gastric, esophageal, breast, and prostate cancers, as well as hematological malignancies and melanoma [104]. These results indicate that different statin categories target different types of cancer. For example, simvastatin exhibits the best efficacy in reducing the risk of GC, whereas it is positively correlated with the risk of pancreatic cancer. As conflicting results were obtained in various clinical observation studies, it is necessary to perform a large-scale prospective trial on the effects of statins in cancer therapy, which will assist physicians in determining the type of statin that is suitable for a specific type of cancer and in understanding the side effects of their use before administration to patients. In addition to further investigating the relationship between the use of statins and GC, the potential applications of statins for the treatment of other types of cancer are worth examining.

## Figures and Tables

**Table 1:** The effects of statins on GC prevention.

Author and Study Year	Study Location	Type of Statin	Effect of Statins on Gastric Cancer	Experimental Studies	Analysis of *H. pylori* Status	Reference
Toyoda, 2009	Japan	Pitavastatin	Pitavastatin was ineffective for chemoprevention of gastric carcinogenesis in gerbils	Rodent models	+	[94]
Chiu, 2011	Taiwan	Lovastatin, pravastatin, rosuvastatin, fluvastatin, simvastatin, and atorvastatin	Any statins are associated with a reduction in gastric cancer risk	Clinical	+	[18]
Lee, 2012	Korea	NA ^†^	The longer prescription of statins, the more reduced risk of gastric cancer	Clinical	+/‒ ^¶^	[27]
Lai, 2013	Taiwan	Lovastatin, pravastatin, rosuvastatin, fluvastatin, simvastatin, and atorvastatin	Simvastatin significantly reduces gastric cancer risk with a dose-response relationship	Clinical	‒	[95]
Lin, 2016	Taiwan	Simvastain and lovastatin	Statins reduce the risk of gastric cancer significantly	Clinical and in vitro	+	[13]
Cheung, 2019	Hong Kong	NA	Statins lower the risk of *H. pylori*-eradicated gastric cancer	Clinical	‒	[96]
You, 2020	Korea	Pravastatin, simvastatin, atorvastatin, cerivastatin, lovastatin, and fluvastatin	Statins decrease gastric cancer incidence in patients with hypercholesterolemia	Clinical	‒	[28]

^†^, not available; ^¶^, including both *H. pylori* infection and non-*H. pylori* infection.

**Table 2:** The effects of statins on GC treatment.

Author and Study Year	Study Location	Type of Statin	Effect of Statins on Gastric Cancer	Experimental Studies	Analysis of *H. pylori* status	Reference
Nam, 2014	Korea	Atorvastatin, rosuvastatin, simvastatin, pitavastatin, fluvastatin, and pravastatin	Statins prescribed more than six month was associated with increased survival	Clinical	‒	[97]
Spence, 2019	UK	NA ^†^	Statins decrease the mortality of gastric cancer	Clinical	‒	[14]
Yang, 2020	Taiwan	NA	Statins increase overall survival of patients with gastric cancer after surgery and adjuvant chemotherapy	Clinical	‒	[15]
Cho, 2021	Korea	NA	Statins lower the mortality of gastric cancer but fail to reduce the incidence	Clinical	‒	[98]

^†^, not available.
